# Percutaneous Coronary Intervention in Transcatheter Aortic Valve Implantation Patients: Overview and Practical Management

**DOI:** 10.3389/fcvm.2021.653768

**Published:** 2021-05-04

**Authors:** Maren Weferling, Christian W. Hamm, Won-Keun Kim

**Affiliations:** ^1^Department of Cardiology, Kerckhoff Heart and Thorax Center, Bad Nauheim, Germany; ^2^German Center for Cardiovascular Research (DZHK), Frankfurt, Germany; ^3^Department of Cardiology, University Hospital of Giessen, Giessen, Germany; ^4^Department of Cardio-Thoracic Surgery, Kerckhoff Heart and Thorax Center, Bad Nauheim, Germany

**Keywords:** TAVI, coronary angiography, PCI, coronary artery disease, acute coronary syndrome

## Abstract

Coronary artery disease (CAD) is present in 40–75% of patients undergoing transcatheter aortic valve implantation (TAVI) for severe symptomatic aortic stenosis. Currently, the indication for TAVI is expanding toward younger patients at lower surgical risk. Given the progressive nature of CAD, the necessity for coronary angiography (CA), including percutaneous coronary intervention (PCI), will subsequently increase as in the future TAVI patients will be younger and have a longer life expectancy. Data on the impact of PCI in patients with severe CAD scheduled for TAVI are controversial, and although European and US guidelines recommend PCI before TAVI, the optimal timing for PCI remains unclear due to a lack of evidence. Depending on the valve type, position, and axial alignment of the implanted device, CA and/or PCI after TAVI can be challenging. Hence, every interventionalist should be familiar with the different types of transcatheter heart valves and their characteristics and technical issues that can arise during invasive coronary procedures. This review provides an overview of current data regarding the prevalence and clinical implications of CAD and PCI in TAVI patients and includes useful guidance for practical management in the clinical routine.

## Introduction

Over the past decade, transcatheter aortic valve implantation (TAVI) has evolved to become the established standard procedure in patients with severe, symptomatic aortic stenosis. Several clinical trials demonstrated non-inferiority of TAVI over surgical aortic valve replacement (SAVR), and consequently, TAVI is now the treatment of choice in patients at intermediate and high surgical risk (1–4). In the recently published PARTNER 3 trial, TAVI was compared with SAVR among patients with low surgical risk and showed even superiority in terms of the composite endpoint of death, stroke, and rehospitalization at 1 year (5). Non-inferiority of TAVI in low-risk patients was also observed in the comparison of a self-expanding valve and SAVR in the randomized EVOLUT Low-Risk trial (6). Data from the NOTION and PARTNER-2A study comparing TAVI with SAVR in patients with low or intermediate surgical risk showed no difference in terms of death and disabling stroke at 5- and 6-year follow-up assessments (7, 8). Recently, the German Society of Cardiology along with the German Society of Thoracic, Heart, and Vascular Surgery released a consensus paper that recommends TAVI in all patients with at least intermediate surgical risk or independent from risk classification when aged ≥75 years; patients below the age of 75 years with low risk should be assigned to a treatment option after individual assessment by a heart team (9).

As the indication for TAVI expands more and more to lower-risk patients with longer life expectancy, the likelihood of requiring coronary angiography (CA), and percutaneous coronary interventions (PCI) for chronic coronary syndromes (CCS) or acute coronary syndromes (ACS) increases. The aim of the present review is [1] to provide an overview of the prevalence and prognostic data of coronary artery disease (CAD) in TAVI patients and [2] to summarize the current knowledge on the management of CA and PCI after TAVI, including guidance for practical routine clinical management. Clinical and practical aspects of PCI in the special subgroup of patients who have undergone a valve-in-valve procedure are beyond the scope of this review and will not be outlined.

## Coronary Artery Disease and PCI: The Dilemma of the Prognostic Role in TAVI Patients

Concomitant CAD is a common finding among TAVI recipients, with a prevalence ranging between 40 and 75% (10). However, the non-uniformity of CAD definition and the variability in the composite of endpoints with limited follow-up duration in most studies impede a reasonable comparison of data: while in some trials CAD was defined according to a history of coronary artery bypass graft (CABG) or prior PCI with no information on persistent relevant coronary artery stenoses (11, 12), in other studies, CAD was defined as either stenosis as high as 50 (13, 14) or 70% in an epicardial vessel (with >50% for left main) (15, 16). Due to this inconsistency, the clinical significance and prognostic role of CAD in patients undergoing TAVI remain unclear and data are still highly variable. In a recently published meta-analysis including more than 8,000 patients from 15 studies undergoing TAVI, preexisting CAD had no impact on 30-day all-cause mortality [odds ratio (OR) 1.07 (95% CI 0.82–1.40); *p* = 0.62], but at 1 year, all-cause mortality was markedly higher in the CAD group [OR 1.21 (95% CI 1.07–1.36); *p* = 0.002] (17). Conversely, another meta-analysis comprising seven studies with 2,472 patients showed that CAD had no impact on mid-term outcome after a median follow-up of 452 days (18). However, in both meta-analyses, CAD severity was not taken into account, e.g., by use of the established SYNTAX score (SS), which is a strong predictor of long-term major adverse cardiovascular and cerebrovascular events (MACCE) and death (19). In yet another meta-analysis, the mere presence of CAD did not affect the outcome, even in multivariate analyses (20). Only a subgroup analysis showed a significantly higher mortality rate at 1 year among patients with a SS > 22, whereas patients with a SS of ≤22 and those who underwent PCI and thereby had a residual SS of <8 had a reduced risk of 1-year all-cause death. Similar results were demonstrated in a retrospective study by Khawaja et al. (15) showing worse 30-day and 1-year outcomes in TAVI patients with a high SS (defined as ≥33) compared with patients with lower SS (<33) (15). Likewise, in an analysis by Stefanini et al. (14), the composite primary endpoint of cardiovascular death, myocardial infarction, and stroke after 1 year occurred significantly more often in the CAD group than in the non-CAD group, and there was a direct relationship between SS and outcome in terms of cardiovascular mortality rate (no CAD 8.6%, low SS 13.6%, high SS 20.4%; *p* = 0.029). A higher residual SS after PCI before or during TAVI had a significant impact on the primary endpoint (14). In contrast, Paradis et al. found no difference in the composite endpoint of all-cause mortality, myocardial infarction, and stroke after 30 days or 1 year between CAD patients stratified according to low (≤22), intermediate (21–30), and high (≥33) SS and patients without CAD undergoing TAVI (13). Finally, the largest study with a multicenter design included 1,270 patients who were stratified into three groups according to their pre-TAVI status: no CAD, non-severe CAD (SS < 22), or severe CAD (SS ≥ 22); those undergoing PCI were further subdivided into “reasonably” incomplete revascularization with a residual SS < 8 and “incomplete” revascularization with a rSS of >8 (31). In both the group with severe CAD and the group with incomplete revascularization, all-cause mortality was higher than that in the other groups after a median follow-up of 1.9 years (31). Table 1 summarizes the key aspects and outcome data of the different studies dealing with the prognosis of CAD in TAVI patients.

**Table 1:** Overview of observational studies on the prognostic impact of CAD in TAVI patients.

**References**	**Sample size**	**CAD definition**	**Prevalence of CAD, *n* (%)**	**SYNTAX score (SS) assessment**	**Outcome data**
Dewey et al. (11)	171	Prior PCI or CABG	84 (49.1%)	N/A	30-day mortality: 13.1% (CAD) vs. 1.2% (no CAD); *p* = 0.002
Ussia et al. (12)	659	Prior PCI or CABG	251 (38.1%)	N/A	12-month mortality:14.5% (CAD) vs. 15.9% (no CAD); *p* = 0.331
Paradis et al. (13)	377	≥50% stenosis in an epicardial vessel ≥1.5 mm in diameter	295 (78.2%)	Low SS (<23), intermediate SS (21–30), high SS (>32); rSS after PCI (<8 vs. ≥8)	Composite primary endpoint (all-cause mortality, MI, and stroke) At 30 days: 13.4% (no CAD) vs. 7.0% (low SS)/10.4% (interm. SS)/9.3% (high SS); *p* = 0.48; also 5.4% for rSS ≥ 8 vs. 0% for rSS < 8; *p* = 0.33 At 1 year: 26.8% (no CAD) vs. 23.3% (low SS)/16.7% (interm. SS)/22.0% (high SS); *p* = 0.6; also 10.8% for rSS ≥ 8 vs. 0% for rSS < 8; *p* = 0.16
Stefanini et al. (14)	445	≥50% stenosis in an epicardial vessel ≥1.5 mm in diameter	287 (64.5%)	Low SS (<23), high SS (≥23), rSS in cases of PCI prior to or during TAVI	Composite primary endpoint (cardiovascular death, MI, and stroke) at 1 year: No CAD: 12.5%, low SS: 16.1%, high SS: 29.6%; *p* = 0.016; No CAD: 12.5%, low rSS (≤14): 16.5%, high rSS (>14): 26.3%; *p* = 0.043
Khawaja et al. (15)	271	≥70% stenosis in an epicardial vessel or ≥50% stenosis for left main or vein graft	93 (34.3%)	Low SS (0–22), intermediate SS (21–30, 32), high SS (≥33)	All-cause mortality: 30-day in no CAD vs. CAD: 7 vs. 7.5%; acc. to SS: 5.2% (low SS) vs. 11.1% (interm. SS) vs. 14.3% (high SS) 1-year in no CAD vs. CAD: 21.5 vs. 23.7%; log-rank *p* = 0.805; acc. to SS: 23.3% (low SS) vs. 22.3% (interm. SS) vs. 57.1% (high SS); log rank *p* = 0.007
Gautier et al. (16)	230	≥70% stenosis in an epicardial vessel or ≥50% stenosis for left main or vein graft	144 (63%)	N/A	Survival at 30 days: CAD (90%) vs. no CAD (85%); *p* = 0.37 Survival at 1 year: CAD (76.4%) vs. no CAD (70.6%); *p* = 0.28
Witberg et al. (31)	1,270	>50% stenosis in at least one epicardial vessel or prior PCI or CABG or MI	453 (36%)	Non-severe CAD (SS 0–22), severe CAD (SS > 22); after PCI: rSS 0–8 or rSS > 8	All-cause mortality at 1 year: Non-severe vs. severe CAD: 26.1 vs. 51.9%; log rank *p* < 0.001 rSS ≤ 8 vs. rSS > 8: 23.2 vs. 45.1%; log rank *p* < 0.001

*CAD, coronary artery disease; TAVI, transcatheter aortic valve intervention; PCI, percutaneous coronary intervention; CABG, coronary artery bypass graft; N/A, not available; MI, myocardial infarction; SS, SYNTAX score; rSS, residual SYNTAX score (after PCI)*.

In summary, the crude classification of “CAD vs. no CAD” in earlier studies is not appropriate to discriminate the underlying risk and leads to inconsistent results regarding the prediction of short- and long-term outcome in TAVI patients. This is predominantly due to the fact that CAD is a highly heterogeneous disease that requires a more differentiated stratification. The more sophisticated approach taken in more recent studies that stratify according to the extent of CAD using the SS, which also reassesses the SS after PCI, has shown greater consistency in the results, with patients with higher SS and higher residual SS after PCI having a predominantly worse prognosis.

## Optimal Timing for PCI: Before, During, or After TAVI?

**Figure 1:**
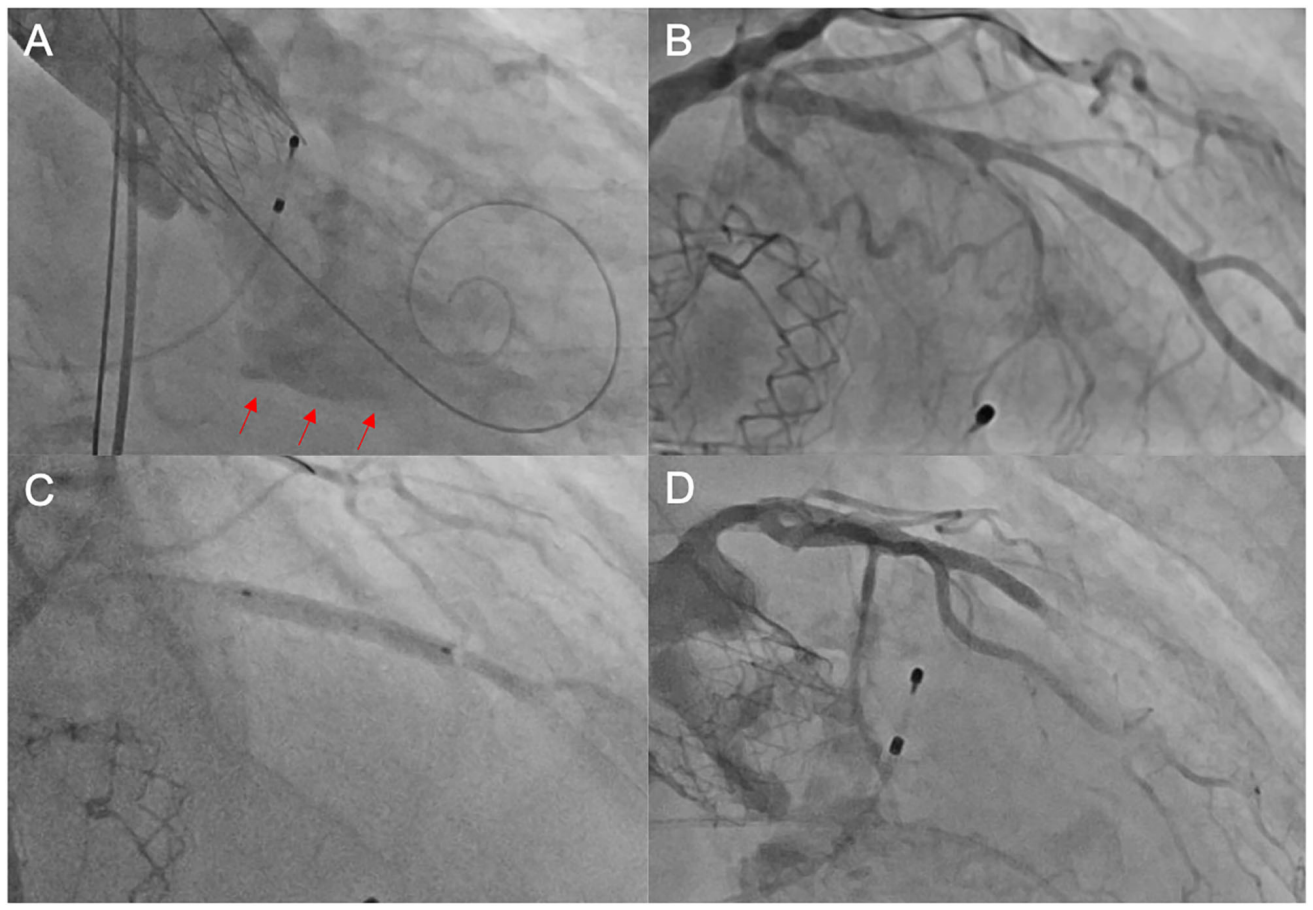
Unsuccessful PCI after TAVI. In this elderly patient, the heart team's decision was to perform TAVI without prior coronary revascularization due to high age and unfavorable morphology. Following TAVI with an ACURATE *neo*™ THV, the patient developed cardiogenic shock due to a combination of relevant paravalvular leakage [red arrows in **(A)**] and presumably untreated coronary artery disease **(B)**. We performed emergent post-dilatation to reduce paravalvular leakage and subsequently carried out coronary intervention of the LAD **(C)** with the implantation of a drug-eluting stent. Thereafter, there was no reflow in the distal LAD **(D)**, and consequently, the patient deceased during the procedure. PCI, percutaneous coronary intervention; TAVI, transcatheter aortic valve implantation; THV, transcatheter heart valve; LAD, left anterior descending artery.

**Figure 2:**
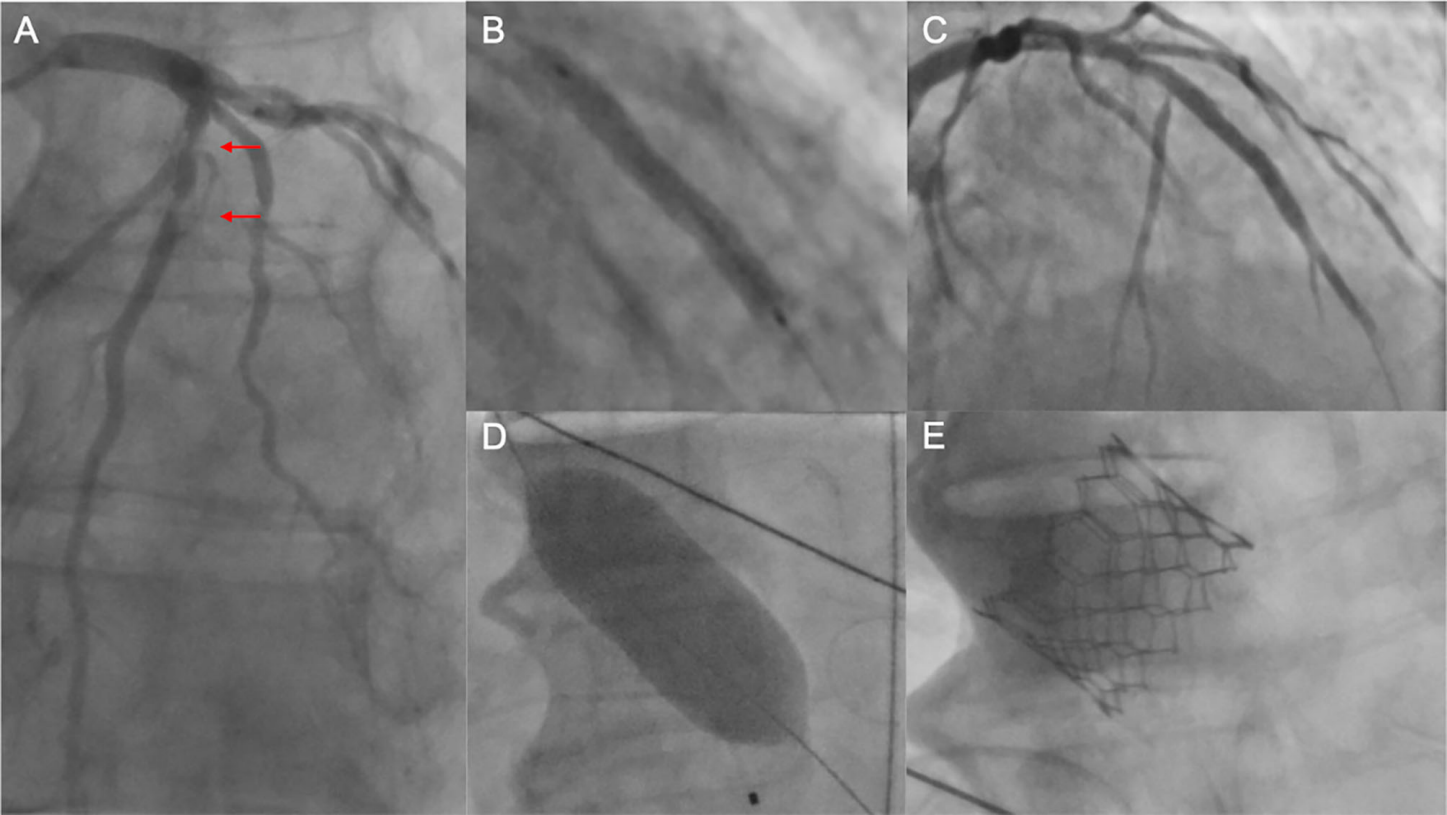
Unsuccessful PCI prior to TAVI. **(A)** Coronary angiography of the left coronary artery with severe tandem stenoses (red arrows) of the LAD in a patient scheduled for TAVI. The decision was made for *ad hoc* PCI. After balloon dilatation, a drug-eluting stent was implanted in the mid-LAD **(B)**. Thereafter, no reflow in the distal LAD was noted **(C)**, and the patient was hemodynamically compromised with the need for cardiopulmonary rescuscitation and emergent balloon valvuloplasty of the aortic stenosis **(D)**. Due to persistent low output and residual high transaortic gradients, the decision was made to perform emergent TAVI using a SAPIEN 3 balloon-expandable device **(E)**. Despite successful TAVI, the patient deceased in the course of multiorgan failure. PCI, percutaneous coronary intervention; TAVI, transcatheter aortic valve implantation; LAD, left anterior descending artery.

Current guidelines recommend CABG in patients planned for SAVR with an epicardial vessel stenosis >70% or left main stenosis >50% with a class I and class IIa level of evidence, respectively (21, 32); however, the recommendations on the timing of PCI in patients with CAD undergoing TAVI are less clear. According to the European guidelines on myocardial revascularization, PCI should be considered in cases with >70% stenosis in the proximal segment of an epicardial vessel in patients planned for TAVI, but the optimal timing for PCI remains “an area of limited evidence” (32). In addition, the European guidelines for the management of valvular heart diseases state that the chronology of interventions in patients planned for TAVI and the need for PCI should be subject to individualized discussion based on the patient's clinical condition and the myocardium at risk (22). In a 2016 consensus statement from the Interventional Section Leadership Council of the American College of Cardiology, PCI before TAVI should be considered for stenoses >70% in major epicardial vessels and the left main coronary artery, as long as the risk of that procedure does not outweigh the benefits (23). But for stenoses at mid or distal parts of the coronary vasculature or stenoses with presumably small areas of ischemia, PCI may be postponed until after TAVI provided that coronary access remains feasible after TAVI. These vague guideline recommendations rest upon scarce data from the evaluation of outcome in relation to PCI timing. Indeed, there are several factors to be taken into account when determining the optimal timing for PCI in patients with high-grade aortic stenosis. Treatment of a relevant epicardial stenosis before TAVI can reduce the ischemic burden, which may become relevant especially in hypotensive phases during the procedure (e.g., during rapid ventricular pacing or flow obstruction during valve implantation) or in the presence of moderate to severe paraprosthetic aortic regurgitation that may enhance coronary ischemia (Figure 1). On the other hand, PCI in a patient with untreated, severe aortic stenosis poses a risk itself, since lesion preparation or possible procedure-related complications like impaired coronary flow during dilatation or coronary artery dissection are less well-tolerated (Figure 2). However, the prospect of coronary access that is not impaired by a valve prosthesis may favor the pre-TAVI approach for PCI (see section below). These considerations notwithstanding, PCI before TAVI requires the need for uninterrupted dual antiplatelet therapy that potentially increases the risk of access site complications. PCI performed during TAVI bears the advantage of using a single access for both PCI and TAVI, but it might be associated with higher radiation doses due to prolonged procedural time and greater amounts of contrast media, increasing the risk of acute kidney injury. Several small sample-sized observational studies investigated the role of PCI before TAVI in terms of outcome: Gasparetto et al. prospectively compared 113 CAD patients (defined as history of PCI/CABG and/or ≥50% stenosis in an epicardial vessel) with 78 patients without CAD undergoing TAVI in terms of 30-day and long-term mortality (mean follow-up time 12.9 months) (24). A portion (20.4%) of the CAD patients underwent PCI before TAVI based on clinical issues such as symptoms, positive ischemia testing, and the myocardium at risk as well as technical feasibility. The authors found no difference in short- (CAD patients 5.7% vs. no-CAD patients 2.9%; *p* = 0.32) and long- term outcomes (15.1 vs. 14.3%; *p* = 0.88) (24). Abdel-Wahab et al. retrospectively compared 30-day and 6-month clinical outcome in 55 patients with PCI before TAVI with 70 patients who had TAVI alone (25). Preprocedural PCI was performed in all cases of stenosis >50% in a major epicardial vessel. After 30 days, neither was there a difference in mortality rates (2% in TAVI and PCI vs. 6% in TAVI alone; *p* = 0.27) nor was there a difference in major bleeding rates or major vascular complications. After 6 months, the outcomes between the groups remained similar (25). A large meta-analysis comprising almost 4,000 patients from nine observational studies analyzed 30-day and 1-year all-cause and cardiovascular mortality in patients receiving PCI before or during TAVI vs. patients who underwent TAVI alone despite the presence of CAD (26). They found a higher all-cause mortality after 30 days and more major vascular complications in the revascularization group, but after 1 year, neither all-cause nor cardiovascular mortality rates were significantly different between the groups. It should be noted that all of these studies were non-randomized and may have been afflicted with a selection bias regarding the decision about which coronary vessel required intervention; furthermore, definitions of CAD used for initial assessment were inconsistent as mentioned above. Studies on the prognostic role of planned PCI intentionally postponed into the period after TAVI are lacking, although several observational studies exist that analyzed the feasibility, technical aspects, and success rates of PCI becoming necessary during the follow-up time after TAVI (see below).

In summary, PCI before TAVI seems to be feasible and may not be associated with worse long-term prognosis compared with no pre-TAVI PCI. Nevertheless, the question of whether patients with significant CAD benefit from PCI before TAVI compared with undergoing no PCI or having PCI after TAVI is still unanswered and can only be addressed by appropriately designed studies, which are currently lacking. The ACTIVATION trial, which compared in a 1:1 randomized fashion pre-TAVI PCI with no PCI in patients planned for TAVI who had a stenosis of at least 70% in a major epicardial vessel, was terminated prematurely due to recruitment issues (27). Generally, each patient planned for TAVI needs to be evaluated by a multidisciplinary heart team, and the decision on possible coronary interventions before, during, or after TAVI needs to be weighed carefully on an individual basis.

## Management of Coronary Angiography and PCI After TAVI

Due to the expansion of TAVI indication toward patients with low surgical risk, the frequency of repeated CA with potential need for coronary interventions in the subgroup of these younger patients with a longer life expectancy will increase. To date, little data exists on the incidence of CA and/or PCI after TAVI outside the periprocedural setting, as a result of ACS, CCS, or other reasons, particularly with respect to long-term follow-up. In the mostly single-center studies available in the literature, the incidence of CA in TAVI patients ranges between 2.5 and 5.3%, with PCI performed in 27–55% of cases (28–30, 33–35) (Table 2). According to Nai Fovino et al., the frequency of CA post-TAVI during a follow-up period of 2.1 years was 5.3% in a single-center cohort comprising 912 patients (30). Among these, 35% of CA was due to ACS, and PCI was performed in 54% of cases. Independent predictors of CA were younger age, previous PCI, and prior CABG. Vilalta et al. reported a 10% ACS rate in a TAVI cohort (*n* = 774) during a median follow-up time of 25 months (34). The majority had a non-ST-segment elevation myocardial infarction (64.1%), and approximately 39% of those patients underwent PCI. In a follow-up study from the SOURCE 3 registry comprising 1,936 patients who were exclusively treated with the SAPIEN 3^TM^ transcatheter heart valve (THV), the rate of CA during a follow-up period of 3 years was 3.5% with a mean time from TAVI to CA of 441 days. Indications for CA were stable CAD (36.8%), NSTEMI (26.5%), and STEMI (11.8%); 69% of those patients underwent PCI (35).

**Table 2:** Overview of studies on coronary angiography (±PCI) after TAVI.

**References**	**Study design**	**Sample size**	**CA/PCI (%) performed**	**THV**	**Indication for CA/PCI**	**Median time from TAVI in days**	**Success rate for PCI (%)**
Perrin et al. (28)	Observational	424	20 (4.7)/11 (55)	Evolut R: 9 CoreValve: 7 Edwards SAPIEN: 2 Evolut Pro: 1	ACS: 8 CCS: 9 Other: 3	464	11/11 (100)
Boukantar et al. (29)	Observational	550	16 (2.9)/7 (43.8)	CoreValve	ACS: 7 CCS: 3 Others: 6	157	6/7 (85.7)
Nai Fovino et al. (30)	Observational	912	48 (5.9)/26 (54)	SAPIEN XT: 21 SAPIEN 3: 15 CoreValve: 6 Evolu Pro2 JenaValve: 2 Lotus: 2	ACS: 17 CCS: 8 Others: 23	769	25/26 (95.2)
Blumenstein et al. (33)	Observational	1,000	31 (3.1)/7 (22.6)	Edwards SAPIEN XT: 16 CoreValve: 10 Symetis ACURATE: 4 Portico: 1	ACS: 4 CCS: N/A Others: N/A	233	7/7 (100)
Vilalta et al. (34)	Observational	78	53 (67.9)/30 (56.6)	N/A	Only ACS patients included	300	N/A
Tarantini et al. (35)	Observational	1,936	68 (3.5)/47 (69.1)	Edwards SAPIEN 3 only	ACS: 26 CCS: 25 Others: 17	441 (mean)	46/47 (97.9)

*CA, coronary angiography; PCI, percutaneous coronary intervention; ACS, acute coronary syndrome; CCS, chronic coronary syndrome; N/A, not available*.

**Figure 3:**
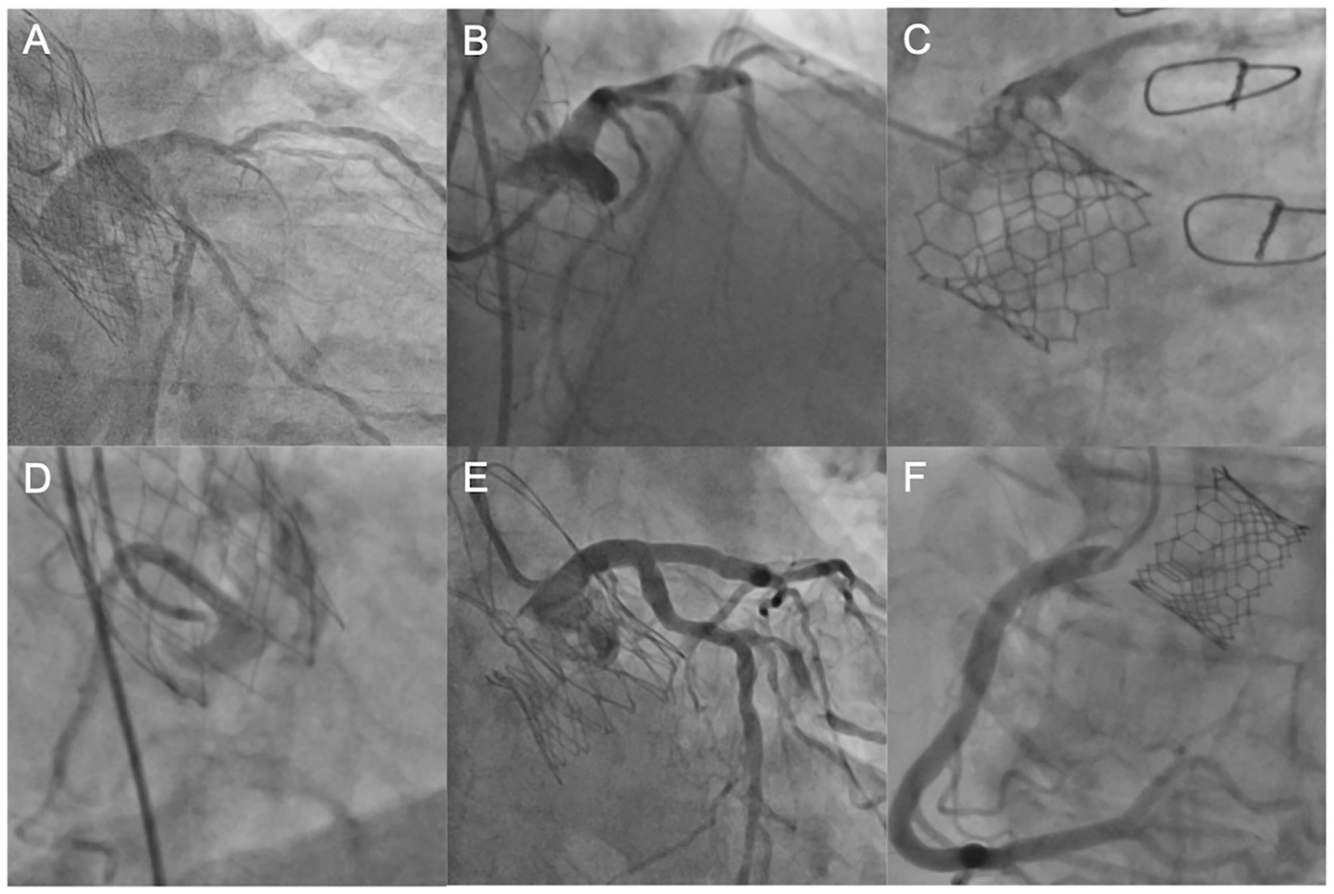
Coronary engagement in different transcatheter heart valves. **(A)** Unselective engagement of the left coronary artery (LCA) using a JL4 diagnostic catheter after TAVI with an Evolut R prosthesis. **(B)** Semi-selective engagement of the LCA using an extra backup catheter (EBU 3.75) in a patient with prior Portico valve. **(C)** Selective, but transprothetic engagement of the LCA with a JL4 catheter following TAVI with a SAPIEN 3 device. **(D)** Unselective catheterization of the right coronary artery (RCA) with a JR4 guiding catheter after TAVI with a Portico device. **(E)** Selective and supra-valvular engagement of a diagnostic JL4 catheter in a patient with prior ACURATE *neo*™ valve. **(F)** Ostial engagement of the RCA in a patient with prior SAPIEN 3 valve. LCA, left coronary artery, JL, Judkins Left, TAVI, transcatheter aortic valve implantation; EBU, extra backup, RCA, right coronary artery.

Coronary interventions after TAVI can be technically difficult. The most challenging aspect of the procedure is the selective cannulation of the coronary ostia, which depends on patient anatomy, valve type and design, and implantation characteristics such as implantation depth and the orientation of the commissural tabs in relation to the ostia (Figure 3). Data on feasibility, success rate, and technical challenges of CA and/or PCI after TAVI are available for a wide range of TAVI prostheses from case reports and smaller case series: from a total of 190 reports, the success rate of selective CA was reportedly 50–100% (36). In the following, we review the literature concerning characteristics and technical aspects of CA (with or without PCI) for the different valve types, e.g., balloon-expandable and self-expanding valves, presenting potential pitfalls as well as our personal experiences.

## Balloon-Expandable Transcatheter Heart Valves

### Coronary Access in Balloon-Expandable Valves

**Figure 4:**
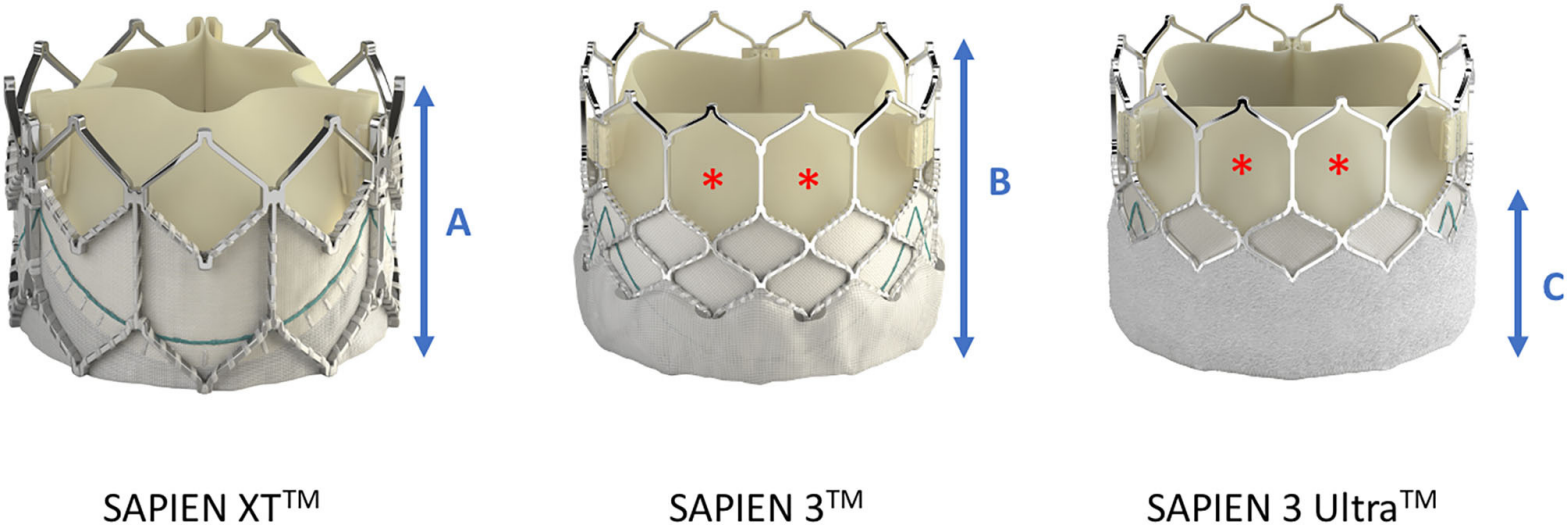
Differences in frame height and cell dimensions between SAPIEN XT^TM^ and SAPIEN 3^TM^/SAPIEN 3 Ultra^TM^. Compared with SAPIEN XT, the frame height **(A,B)** of SAPIEN 3^TM^ and SAPIEN 3 Ultra^TM^ is taller, potentially leading to more interference with the coronary ostia, but the cells in the upper row are bigger thereby facilitating coronary access (red asterisk). The newer SAPIEN 3 Ultra^TM^ retains the SAPIEN 3^TM^ stent frame design, but its outer skirt is 40% higher to provide a better sealing **(C)**. Image courtesy of Edwards Lifescience Corporation.

The feasibility of accessing the coronary ostia and performing PCI after implantation of balloon-expandable THVs (SAPIEN™, Edwards, Irvine, California, USA) has been shown in several observational studies (33, 37, 38). In general, coronary cannulation in patients with balloon-expandable valves is technically less challenging than with self-expanding valves, mainly due to the lower stent frame that in most cases does not protrude over the coronary orifice. However, there is a trend toward using higher implantation depth of the valve, as data over the last few years have consistently shown that a lower device position increases the need for permanent pacemaker implantation (39, 40). In addition, the newer generations of balloon-expandable THVs, e.g., the SAPIEN 3™ and SAPIEN 3 Ultra™, feature a taller stent frame than the precursor SAPIEN XT™, thus increasing the probability of partially or totally covering the coronary ostia (see Figure 4). On the other hand, the cells in the upper row have a 38% larger area than the SAPIEN XT™, which facilitates the engagement of the catheter through the struts and possibly compensates for the higher rate of supra-ostial positioning (41). Compared to the SAPIEN 3™, the newer model SAPIEN 3 Ultra™ (received CE mark approval in November 2018) features a 40% higher outer skirt made from a textured polyethylene terephthalate (PET), which is supposed to facilitate the healing process and improve the sealing. Faroux et al. analyzed angiographic and computed tomography data from a total of 553 patients who underwent successful implantation of Edwards SAPIEN XT™ and SAPIEN 3™ in terms of the position of the THV in relation to the coronary ostia (42, 43). They found a complete coverage of the left main ostium in a total of 27% of cases, with a significantly higher proportion when the newer-generation SAPIEN 3™ was implanted (43 vs. 12%; *p* < 0.001). Notably, 10% of patients underwent CA (with or without PCI) after TAVI, and no differences in CA performance and/or PCI results in relation to valve position were found. Ferreira-Neto et al. compared the feasibility and success rate in 41 patients with balloon-expandable valves (SAPIEN™ and SAPIEN XT™) who underwent CA and/or PCI before and after TAVI during a mean follow-up period of 2 years (38). Independent of the position of the valve (in the PCI group, 23% at the supra-ostial and 38.5% at the ostial or infra-ostial level), no differences in terms of procedural factors including arterial access site, number and choice of catheters, procedural duration, fluoroscopy time, contrast agent volume, and successful selective coronary injection were found between pre- and post-TAVI CA (38).

Another aspect to be taken into account for CA in post-TAVI patients is a possible commissural misalignment in relation to the coronary ostia. In contrast to SAVR, where the valve and its commissural tabs can be aligned with respect to the position of the native coronary orifices, proper alignment is much more difficult to achieve for TAVI. Tang et al. examined the anatomic relation between neo-commissures and coronary ostia in 70 patients with CT scans available before and after TAVI (*n* = 53 with either SAPIEN™, SAPIEN XT™, or SAPIEN 3™; *n* = 17 with Evolut R™) (44). In over 50% of the cases, they found a severe overlap between the neo-commissural tabs and either the left coronary artery (LCA) orifice or right coronary artery (RCA) or both. There was no difference regarding the frequency of overlap between balloon-expandable and self-expanding THVs. The clinical impact of such a close anatomic proximity is currently unclear, however, and the authors did not provide any information on possible interference with catheter engagement (44).

### Engagement of Guiding Catheters in Balloon-Expandable Valves

As in most cases the outflow aspect of the THV stent frame will be located below the coronary ostia, coronary engagement with guiding catheters commonly will be uncomplicated and may not differ from the situation with a native valve. If the stent frame protrudes over the coronary orifice, the intubation of the guiding catheter must be performed through the stent struts near the coronary entrance, ideally in a coaxial orientation. If the coronary ostium is covered by the skirt or a commissural tab, the cannulation should be performed across the strut that is most adjacent to the coronary ostium. For guiding catheter engagement of the LCA, a Judkins Left (JL) 4 or JL 3.5 as well as an extra backup (EBU) catheter 3.5/3.75 can be used; for the RCA, usually a Judkins Right 4 (JR4) is appropriate.

## Self-Expanding Transcatheter Heart Valves

### Coronary Access in Self-Expanding Valves: CoreValve Evolut-R/Pro™

**Figure 5:**
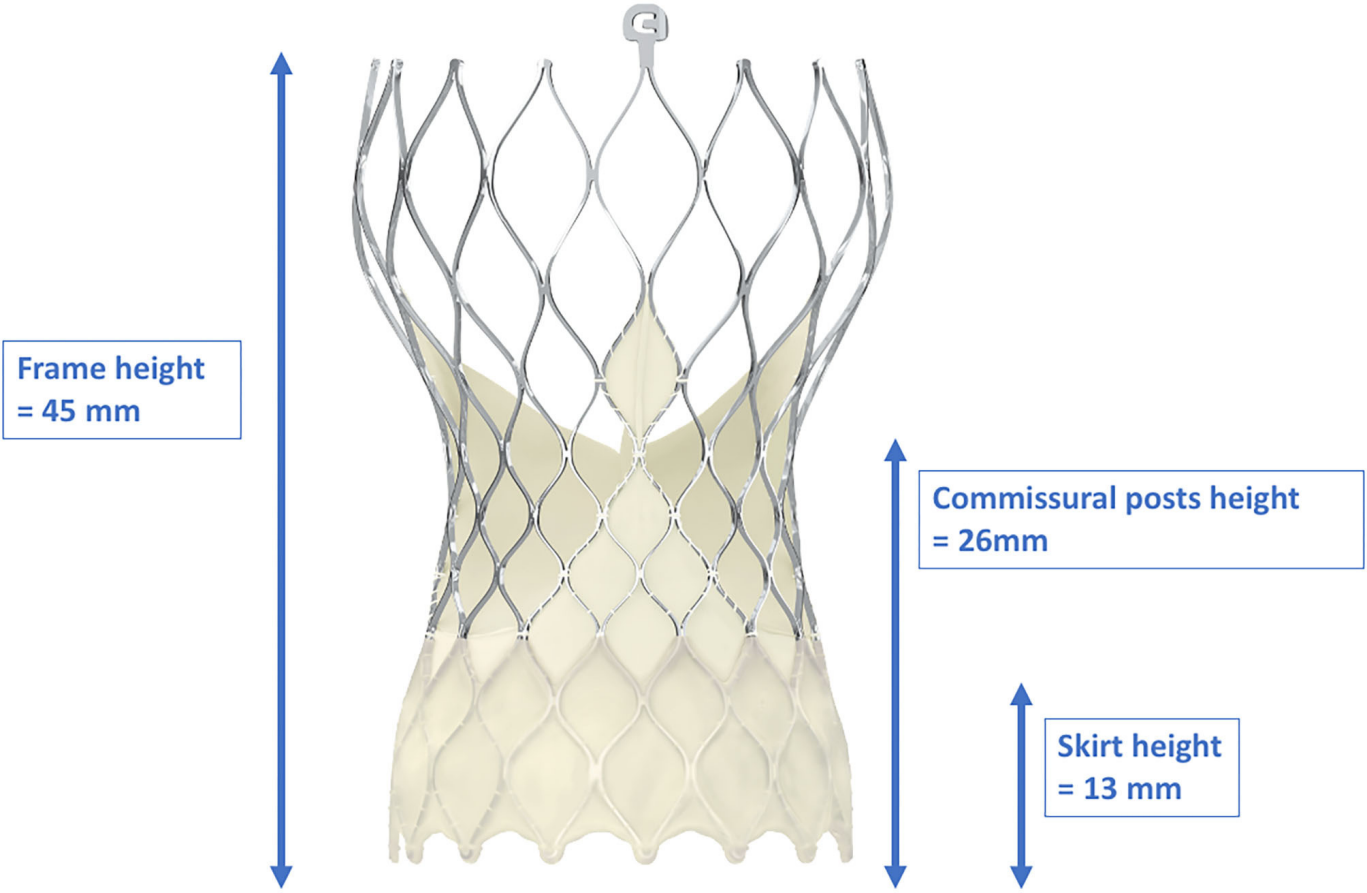
Dimensions of the frame, skirt, and commissural post heights of the CoreValve Evolut-Pro^TM^. With a frame height of 45 mm, the CoreValve Evolut-Pro^TM^ inevitably exceeds the coronary ostia. Due to the “waist” of the stent frame, enough space between the coronary ostia and the valve is provided. With kind approval by Medtronic (Dublin, Ireland).

**Figure 6:**
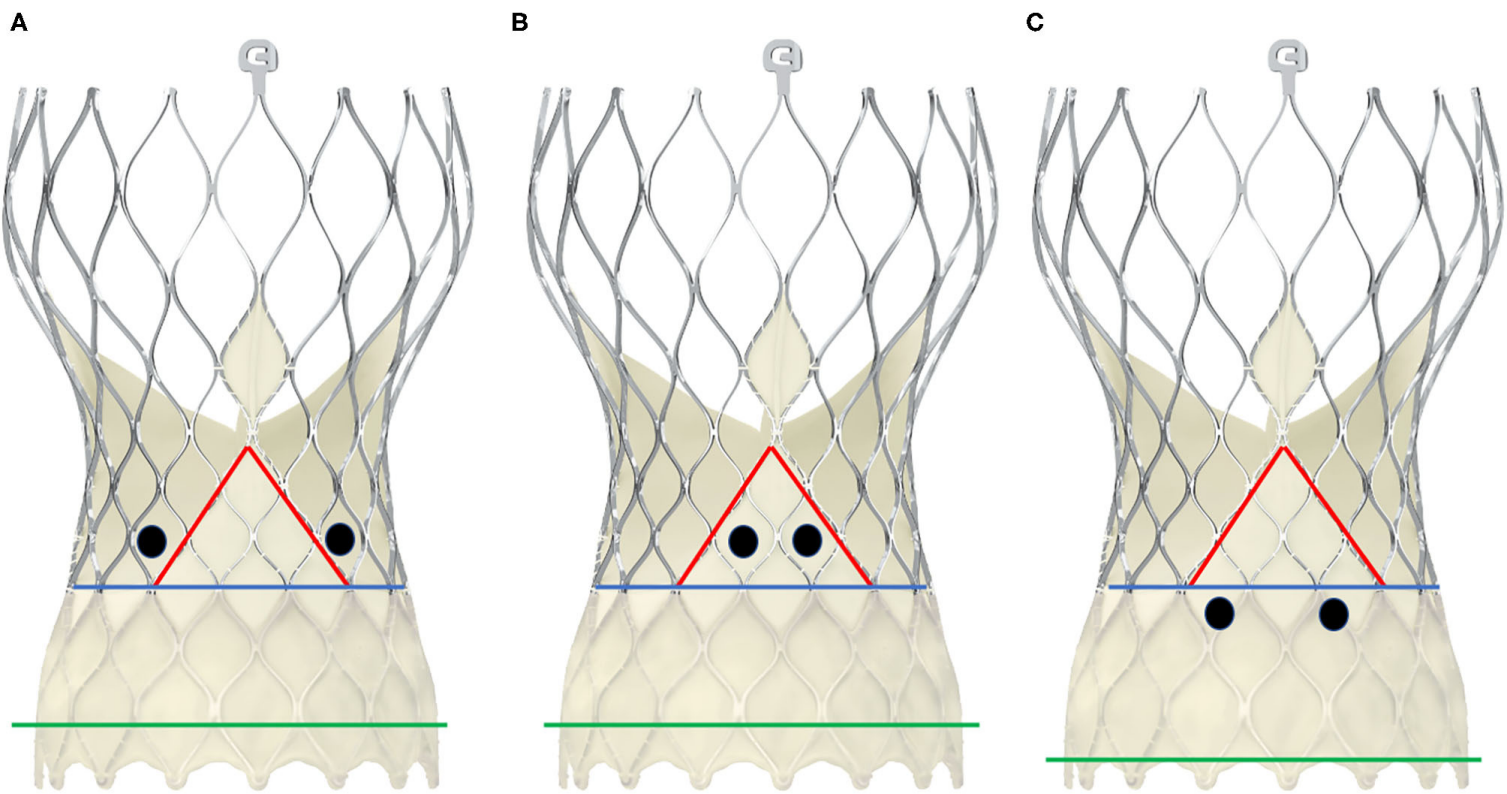
Schematic presentation of different positions of the coronary ostia in relation to the skirt and the commissural posts in CoreValve Evolut-Pro^TM^. The black dots represent two examples of positionings of the coronary ostia. The green line depicts the annular plane, the blue line represents the upper limit of the sealing skirt, and the red line marks the course of the skirt rising up with the commissural post. In scheme **(A)**, the coronary orifices are beyond the skirt and the commissural posts, so that a coaxial intubation is likely to be achieved. In scheme **(B)**, the coronary ostia are in front of the commisural post, so the cannulation must be performed through a diamond adjacent to it. Scheme **(C)** shows an example of low-lying coronary ostia and low implantation depth, with the sealing skirt facing the coronary ostia. Coronary engagement should be performed from a diamond above.

In contrast to balloon-expandable valves, the proper engagement of the coronary ostia in patients with self-expanding valves, e.g., the CoreValve™ (Medtronic Inc., Minneapolis, Minnesota, USA), is more challenging, and semi-selective or even unselective angiograms as well as unsuccessful PCIs are much more common than in cases with balloon-expandable valves (29, 33, 45). There are several reasons for coronary access difficulties with these THVs. The Evolut-R/Pro™ is a supra-annular valve with a frame length of 45–46 mm that invariably extends over the coronary ostia. It consists of an inflow portion with high radial force that retains the valve in the annular plane, a concave waist that leaves enough space between the frame and the coronary ostia, and an outflow segment situated in the ascending aorta (41) (Figure 5). Yudi et al. recently published a sophisticated report on the geometric interaction between the Evolut-R/Pro™ and the coronary ostia (41). The skirt height is 13 mm (14 mm in the 34-mm Evolut-R/Pro™), which can lead to interference with the coronary ostia when the implantation depth is high and/or the height of the native coronary ostia is low (<10 mm). Due to the higher rates of permanent pacemaker implantation demonstrated for the CoreValve™ (46), the operators tend to implant the valve as high as reasonably possible to reduce the risk of conduction disturbances (47), but this approach increases the risk of the skirt overlapping the ostia. The recommended implantation depth is 3–5 mm (41). In contrast to balloon-expandable valves, the CoreValve Evolut-R/Pro™ can be recaptured and repositioned during the deployment process, allowing for optimization of the THV's alignment in relation to the annular plane and the coronary orifices. However, the neo-valve itself can interfere with the ostia: Couture et al. described a supra-ostial and ostial position of the neo-valve in 3 and 12% of cases, respectively, with an implantation depth of ≤6 mm as the strongest predictor for this positioning (43). As for the balloon-expandable valves, Yudi et al. noted that a possible interaction of the neo-commissures of the self-expanding valve with the native coronary arteries could further aggravate proper engagement of the guiding catheter (41); in the case of the Evolut-R/Pro™, although the sealing skirt is only 13-mm (14 mm) high, at the commissural insertion point it rises up to 26 mm, making an interference with the coronary ostia more likely and a coaxial engagement of the catheter more challenging. In contrast to SAVR, as mentioned above, during the TAVI procedure, it is not possible to align the commissural posts of the THV with the native commissures. Figure 6 depicts three different scenarios for the relationship between the coronary ostium and the skirt and the stent frame struts of the Evolut-R/Pro™. Finally, the more shallow the sinus of Valsalva, the lesser the space that lies between the ostia and the stent frame, making it more difficult to manipulate the catheter and properly intubate the orifice. This is even more challenging when the native valve leaflets captured between the stent frame and the sinus of Valsalva are heavily calcified and bulky, thereby reducing the space for manipulation of the catheter even more (41).

### Engagement of Guiding Catheters in Self-Expanding Valves: CoreValve Evolut-R/Pro™

**Figure 7:**
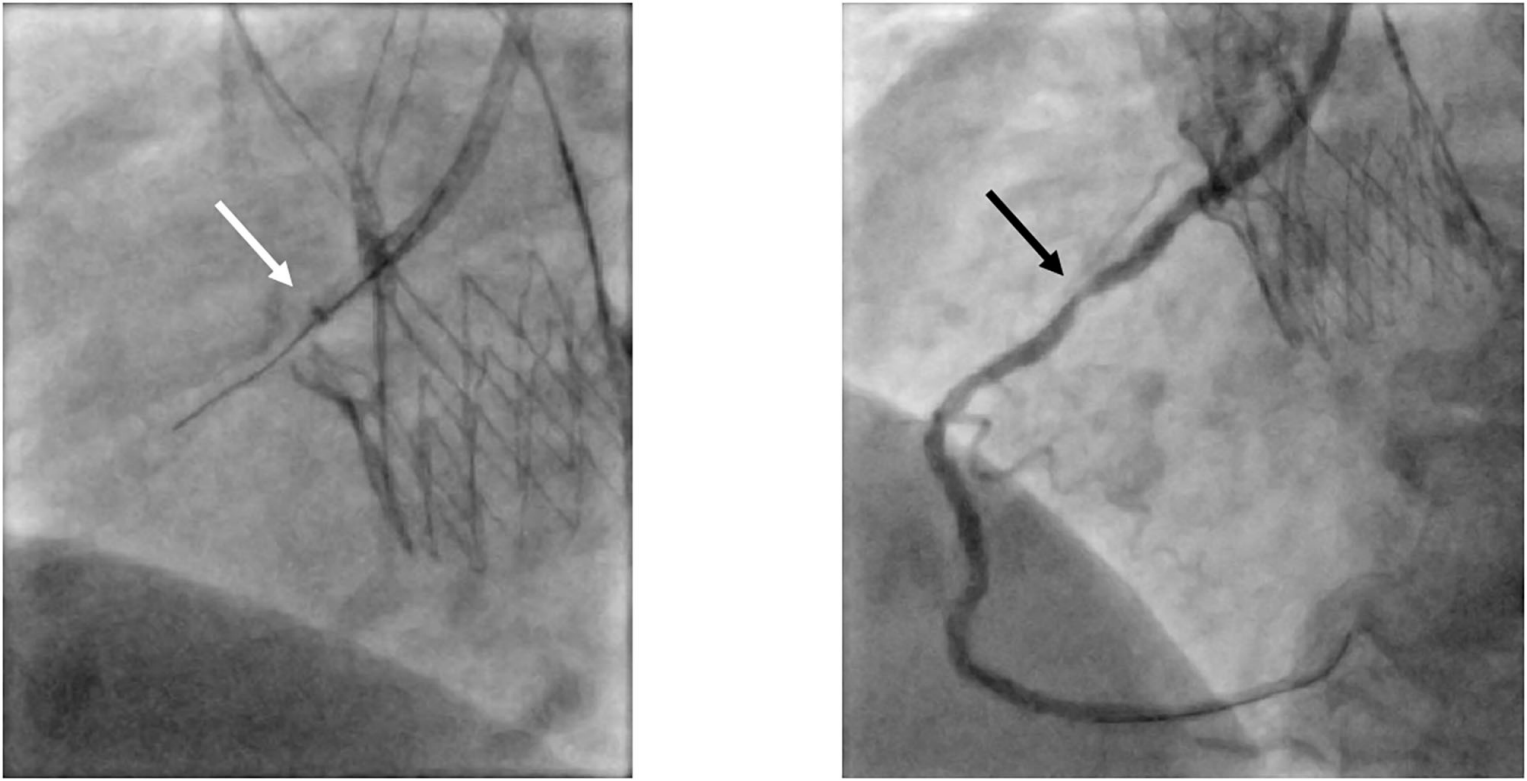
Selective engagement of the RCA via a guiding catheter extension. In this patient presenting with acute coronary syndrome with the culprit lesion in the mid-section of the RCA (black arrow), proper engagement of the RCA with the guiding catheter JR4 was not possible. Advancing a guiding catheter extension (white arrow; Guidezilla^TM^, Boston Scientific, Marlborough, Massachusetts, USA) led to successful selective cannulation. RCA, right coronary artery; JR, Judkins Right.

Since the stent frame of the CoreValve™ prosthesis always extends beyond the coronary ostia, the guiding catheter is required to pass through the stent struts to successfully intubate the coronary arteries. If possible, the intubation of the coronary ostia should be performed coaxially through a stent cell at the level of the coronary orifice. Due to the “waist” of the CoreValve™, which measures 20–24 mm depending on the valve size, the space to manipulate the catheter is clearly smaller than the native aortic root dimensions. Hence, smaller guiding catheters for the LCA, like JL 3.5 or even JL 3.0 instead of JL 4.0, might be a better choice for successful engagement. This recommendation is in line with that of Yudi et al. (41) and Blumenstein et al. (33), although in the latter retrospective analysis of technical aspects of CA (with or without PCI) in TAVI patients, only diagnostic CAs were performed for CoreValve™ patients (33). For the RCA, on the other hand, a JR 4 guiding catheter is mostly sufficient, although, when the sinus of Valsalva is wide and there is more space between the stent frame and the coronary orifice, a longer-tipped guiding catheter such as an Amplatzer right (AR) 2 or even a multipurpose catheter is preferred. In cases with superimposed neo-commissures or even when portions of the THV skirt extend in front of the ostia, intubation in a coaxial manner would most probably lead to insufficient cannulation; thus, an intubation from above through a diamond cell above the ostium is recommended. Caution is warranted when using EBU catheters for the LCA. Harash et al. reported a case where an EBU 3.5 became entrapped within the stent frame of an Evolut™ prosthesis after successful PCI of the LAD with subsequent dissection of the left main following attempts to disengage the catheter (45). The authors concluded that catheter retrieval failed due to the sharp angle between the tip of the guiding catheter and the stent frame as well as the engagement from a diamond below the ostium. If problems occur with proper engagement of the guiding catheter, a coronary wire advanced in the coronary artery can be used to rail the catheter into the ostium. In addition, a small balloon (e.g., 2.0/12 mm) can be positioned in the left main to guide the catheter in (balloon-assisted tracking). The balloon can also be carefully inflated in a more peripheral position in the coronary vessel to further “pull” the catheter into the ostium (anchoring balloon technique) (28). Of course, care should be taken during this maneuver to avoid dissections. As a bail-out strategy, an extension catheter (e.g., GuideLiner™, Vascular Solution, Minneapolis, Minnesota, USA or Guidezilla™, Boston Scientific, Marlborough, Massachusetts, USA) can be advanced cautiously into the coronary ostium to rail the guiding catheter into the correct position (mother-and-child). In some cases, the guiding catheter might still not be advancing into the coronary ostium, either due to unfavorable interference of the stent frame's strut and/or to the lack of co-axial alignment of the guiding catheter to the ostium. Here, the guiding catheter extension can be used for the selective cannulation and the procedure can be performed solely through the guiding catheter extension device properly inserted into the coronary ostium. In addition, as mentioned above for engagement of the guiding catheter, “balloon-assisted tracking” or the “anchoring-balloon technique” can also be applied for the guiding catheter extension to support the selective engagement of the coronary ostium. Figure 7 shows a guiding catheter extension inserted into the RCA ostium with successful PCI. The maneuvers mentioned above can be performed for all types of THVs when there are problems with proper engagement of the guiding catheter.

### Coronary Access in Self-Expanding Valves: Acurate *neo*™

**Figure 8:**
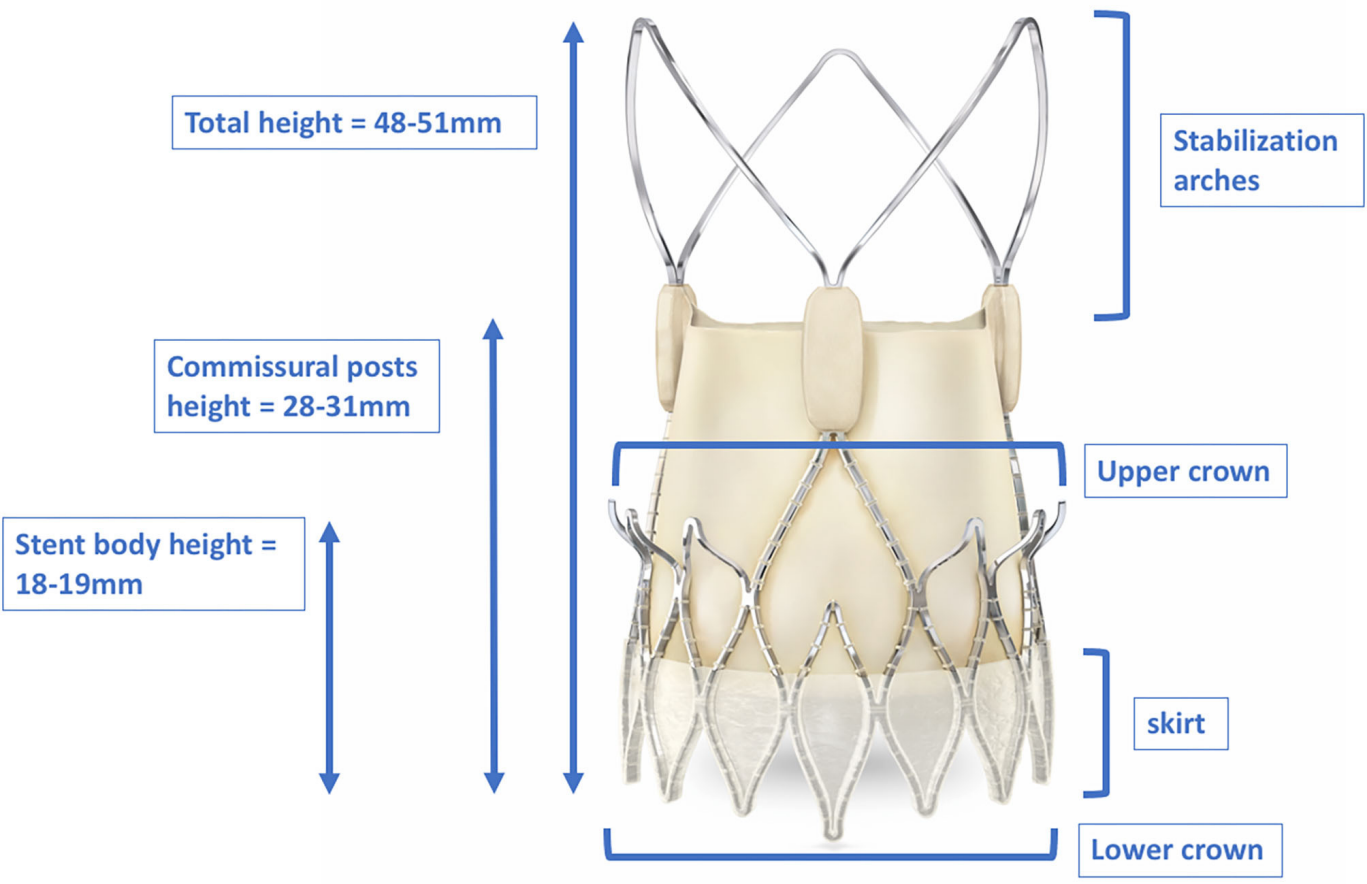
Dimensions of the total height, stent body height, and commissural post height of the ACURATE *neo*^TM^. The upper crown of the stent body captures the native leaflets and anchors the device supra-annually. The stabilization arches provide axial self-alignment within the ascending aorta. With kind approval by Boston Scientific (Marlborough, Massachusetts, USA).

The ACURATE *neo*™ THV (Boston Scientific, Marlborough, Massachusetts, USA) (Figure 8) is a self-expanding valve consisting of an upper crown for supra-annular anchoring and capturing the native leaflets, a lower crown with minimal protrusion into the left ventricular outflow tract, and stabilization arches for axial self-alignment within the ascending aorta (48). The device received CE certification in 2014. Currently, it is only available in Europe, South America, Canada, and the Asia-Pacific region; approval by the Food and Drug Administration (FDA) of the United States is pending.

Data regarding CA and/or PCI for this THV are scarce. With its stabilization arches extending up into the ascending aorta, the device definitely protrudes over the coronary ostia, thus possibly impeding catheter engagement. In addition, as for the CoreValve™, the commissural post height (28–31 mm, depending on the valve size) is quite high and a commissural tab might randomly be positioned in front of the coronary ostia during the implantation process. Thus far, only one study has provided retrospective data on CA and PCI in patients with this type of device: Blumenstein et al. reported on four patients with the precursor model Symetis ACURATE™ (33). Selective CA was only possible in two patients; in the other two, semi-selective CA was performed that still allowed proper evaluation of the coronary arteries. One patient underwent PCI of the left anterior descending artery (LAD), which was achieved by using an AL2 guiding catheter. However, intubation with that guiding catheter was initially semi-selective, and subsequent guidewire advancement led to proper engagement and successful PCI (33).

## Conclusion

TAVI is increasingly being performed in younger patients with low surgical risk and longer life expectancy. As CAD is a progressive disease, coronary interventions in TAVI patients will become more common due to the widening indication for TAVI. Knowledge of the different THVs, their structural and functional characteristics, and their possible interference with the coronary ostia is of paramount importance for operators. More research efforts are warranted in order to better define proper and safe techniques performing CA and PCI in TAVI patients.

## Author Contributions

MW: drafting of the manuscript. CH: critical revision of the manuscript and final approvement. W-KK: drafting of the manuscript, critical revision of the manuscript, and final approvement.

## Conflict of Interest

CH is an advisory board of Medtronic. W-KK received proctor and/or speaker fees from Abbott, Boston Scientific, Edwards Lifesciences, Medtronic, and Meril Life Sciences. The remaining author declares that the research was conducted in the absence of any commercial or financial relationships that could be construed as a potential conflict of interest.
